# Clinical impact of upper gastrointestinal endoscopy in critically ill patients with suspected bleeding

**DOI:** 10.1186/s13613-018-0423-5

**Published:** 2018-07-04

**Authors:** Sylvain Jean-Baptiste, Jonathan Messika, David Hajage, Stéphane Gaudry, Julie Barbieri, Henri Duboc, Didier Dreyfuss, Benoit Coffin, Jean-Damien Ricard

**Affiliations:** 1 Medico-Surgical Intensive Care Unit, AP-HP, Hôpital Louis Mourier, 178 rue des Renouillers, 92700 Colombes, France; 2 IAME, UMR 1137, INSERM, 75018 Paris, France; 3 IAME, UMR 1137, Univ Paris Diderot, Sorbonne Paris Cité, 75018 Paris, France; 4 Département de Biostatistiques, Santé Publique et Information Médicale, AP-HP, Hôpital Pitié-Salpêtrière, 75013 Paris, France; 5 Univ Pierre et Marie Curie, Sorbonne Universités, 75013 Paris, France; 6 ECEVE, U1123, CIC-EC 1425, INSERM, 75010 Paris, France; 7 ECEVE, UMRS 1123, Univ Paris Diderot, Sorbonne Paris Cité, 75010 Paris, France; 8 Gastroenterology Unit, AP-HP, Hôpital Louis Mourier, 178 rue des Renouillers, 92700 Colombes, France; 9 Univ Paris Diderot, Sorbonne Paris Cité, 75018 Paris, France

**Keywords:** Upper gastrointestinal endoscopy, Intensive care unit, Profitability

## Abstract

**Background and Aims:**

Upper gastrointestinal endoscopies’ (UGE) profitability is undisputable in patients admitted for an overt upper digestive tract bleeding. In critically ill subjects admitted for other causes, its performances have scarcely been investigated despite its broad use. We sought to question the performance of bedside UGE in intensive care unit (ICU) patients, admitted for another reason than overt bleeding.

**Methods:**

This was a six-year (January 2007–December 2012) retrospective observational study of all UGE performed in a medico-surgical ICU. Exclusion of those performed: in patients admitted for a patent upper digestive bleeding; for a second-look gastroscopy of a known lesion; as a planned interventional procedure. Main demographic and clinical data were recorded; UGE indication and profitability were rated according to its findings and therapeutic impact. Operative values of the indications of UGE were calculated. This study received approval from the Ethics Committee of the French Society of Intensive Care (n° 12-363).

**Results:**

Eighty-four patients (74% male, mean age 61 ± 14 years) underwent a diagnostic UGE, all for a suspected upper digestive tract bleeding. The main symptoms justifying the procedure were anemia (52%), digestive bleeding (27%), vomiting (15%), hemodynamic instability (3%) and hyperuremia (3%). The profitability of UGE was rated as major (*n* = 5; 5.8%); minor (*n* = 34; 40.5%); or null (*n* = 45; 53.6%).

**Conclusions:**

When ICU admission is not warranted by a digestive bleeding, UGE has limited diagnostic and therapeutic interest, despite being often performed.

## Background

Bedside upper gastrointestinal endoscopy (UGE) is a procedure frequently performed in critically ill patients admitted to the intensive care unit (ICU). It has both a diagnostic (macroscopic examination of the lesions and biopsy sampling) and a therapeutic role (hemostatic vasoconstrictor injection, clipping, ligation of esophageal varices, etc.).

Its performance is well demonstrated for the management of patients admitted for upper digestive tract bleeding [1–9]. Nevertheless, apart from this specific context, a bedside UGE is also frequently performed in patients admitted for another reason, in which an upper digestive bleeding suspicion is raised during the course of ICU stay. The reasons for such suspicion might be the occurrence of exteriorized bleeding, an acute anemia, an hemodynamic instability or an hyperuremia without renal failure [10–12].

The performance and added value of UGE in this indication are much more debated [10, 13–16]. Indeed, critically ill patients often present with complex medical situations, many reasons to account for these signs (such as bleeding from another site, inflammatory anemia, sepsis and acute kidney injury). Furthermore, the discovery of a mucosal lesion might not require any endoscopic nor pharmacological treatment (gastritis, esophagitis, nasogastric tube-associated ulcerations) [17, 18] and hypothetic benefits of this procedure must be weighed against its inherent costs and risks [19]. We therefore questioned the performance of a bedside UGE in critically ill patients, admitted for another reason than upper digestive bleeding in the ICU. Our hypothesis was that a majority of procedures, performed in a general ICU population to confirm or exclude a GI bleeding, would only find nonspecific, ICU-associated lesions that would not significantly influence the patients’ management.

## Patients and methods

We conducted a retrospective, monocenter study, in a single teaching-based, 12-bed medico-surgical ICU, of all UGE performed between January 2007 and December 2012. The Ethics Committee of the French Society of Intensive Care (SRLF) approved the study (n° 12-363). An informed consent was waived due to the retrospective design of the study.

All the patients who underwent a gastroscopy were identified through the endoscopy unit’s database, in which all UGEs performed in our institution are registered. All the patients who had an UGE performed during their ICU stay were included, and their whole medical records were analyzed. Excluded patients were those admitted for a patent upper digestive bleeding or those who underwent a second-look gastroscopy of a known lesion or for a planned interventional procedure (such as a gastric tube or esophageal prosthesis placement). When a patient underwent several procedures, only the first one was taken into account.

Data collected were age, gender, the reason for ICU admission, the Sequential Organ Failure Assessment (SOFA) score [20] at ICU admission and the number of organ failures according to the individual organ score of the SOFA score [20]. We also recorded the symptoms and conditions that motivated the UGE, its findings and the subsequent procedures performed if any. Vital status at ICU discharge was also recorded.

UGE was performed by a gastroenterologist (in most cases by a senior physician and in rare instances, by a trainee under a senior’s supervision) with standard Fuji video gastroscope. It was performed under general anesthesia with orotracheal intubation or under sedation in patients with no previous intubation. If gastric hemorrhage was suspected, a 2.5 mg/kg IV infusion of erythromycin was performed 30–60 min before endoscopy.

For each UGE, its profitability was rated. It was considered as “major” if it allowed a hemostatic procedure or the diagnosis of a cancer; “minor” if it allowed a diagnosis of a peptic disorder, which could be pharmacologically treated; or “null” if it was normal or if the findings had no therapeutic consequence.

Continuous data are presented as mean and standard deviation unless otherwise indicated. Dichotomous data are presented as number and percentage. For each reason motivating the UGE, we calculated the operative values (sensibility, specificity, positive and negative predictive value) associated with a major or minor profitability of the UGE. The analyses were performed with R version 3.2.0.

## Results

### Patient demographics and characteristics

**Fig. 1:**
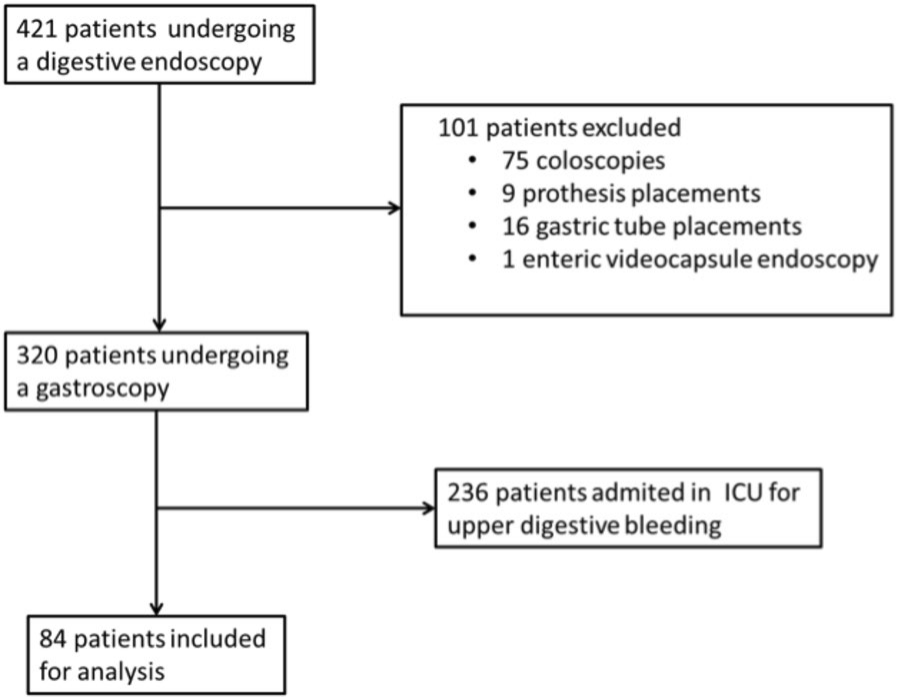
Patient flowchart. During the study period, 3352 patients had been admitted in our ICU, and 320 underwent an upper gastrointestinal endoscopy during their ICU stay, among whom 84 had had not been admitted for upper digestive bleeding

Patients’ flowchart is shown in Fig. 1, and patients’ characteristics are summarized in Table 1. Among 3352 ICU admissions, 84 patients (74% male, mean age 61 ± 14 years) had not been admitted for upper digestive bleeding and underwent a diagnostic UGE during their ICU stay, after a mean of 13 ± 16 days of ICU admission. Main reason for ICU admission was sepsis (81%), 38% were surgical patients, and 7% were admitted in ICU following a gastroesophageal surgery. A multiorgan failure was present in most of them, as 92% received invasive mechanical ventilation and 62% had vasopressors. The mean SOFA score at admission was 7.7 ± 3.7. In our cohort, ICU mortality was 29%.

**Table 1:** Characteristics of the 84 critically ill patients undergoing a bedside upper gastrointestinal endoscopy

Patients’ characteristics. *n* = 84	
Age (years)	61.7 ± 14
Male sex	62 (74%)
Medical patients	52 (62%)
Surgical patients	32 (38%)
Esogastric surgery	6 (7%)
SOFA score	7.7 ± 3.7
Mechanical ventilation	77 (92%)
Vasopressor	52 (62%)
Acute kidney injury	50 (60%)
Sepsis	68 (81%)

Data are expressed as mean ± SD, or *n* (%); SOFA: Sequential Organ Failure Assessment SOFA scores can range from 0 (no organ failure) to 24 (most severe level of multiorgan failure)

### UGE indication

The reasons for performing the UGE are shown in Table 2. In every case, an upper bleeding was suspected. The main symptoms justifying the procedure according to the attending physician were acute anemia (52%), digestive bleeding (27%), vomiting (15%), hemodynamic instability (3%) and hyperuremia (3%).

**Table 2:** Reasons for performing the upper gastrointestinal endoscopy in the 84 critically ill patients

Acute anemia	50 (60%)
Digestive bleeding	26 (31%)
Vomiting	14 (17%)
Hemodynamic instability	3 (4%)
Hyperuremia	3 (4%)

One patient could have various reasons for performing the upper gastrointestinal endoscopy. Data are presented in *n* (%)

### UGE findings

The findings of the UGE are shown in Table 3. It was considered normal in 30% of those (*n* = 25). Among abnormal findings, the most frequent were nasogastric tube erosions (*n* = 18), peptic gastritis or esophagitis (*n* = 14), peptic gastric ulcer (*n* = 13), esophageal candidiasis (*n* = 7), esogastric varices (*n* = 4). According to our pre-specified classification, we considered that 5 UGE had a major profitability (5.8%) and 34 (40.5%) had a minor profitability, and for 45 (53.6%), this profitability was null.

**Table 3:** Findings of the 84 upper gastrointestinal endoscopy performed

Normal	25 (30%)
Esophagitis or gastritis	14 (17%)
Nasogastric tube erosion	18 (21%)
Peptic ulcer	13 (15%)
Esophagogastric varices	4 (5%)
Amyloidosis	1 (1%)
Esophageal candidosis	7 (8%)
Cancer	2 (2%)

Data are presented as *n* (%)

### Diagnostic and predictive value

Sensibility, specificity, positive and negative predictive value (PPV and NPV) of the symptoms and conditions that motivated the procedures are shown in Table 4. Hemodynamic instability had a PPV of 100% but was present in only 3 patients and always associated with at least another sign (drop in hemoglobin in 3 and overt digestive bleeding in 2). The second best PPV was 66.7% for hyperuremia. It has to be noted that that acute anemia and digestive bleeding have not a high PPV (61.5 and 48%, respectively).

**Table 4:** Diagnostic and predictive values of upper gastrointestinal endoscopy in critically ill subjects

	Sensibility	Specificity	PPV	NPV
Esogastric surgery	7.7	93.3	50.0	53.8
Acute kidney injury	46.2	28.9	36.0	38.2
Coagulopathy	20.5	80.0	47.1	53.7
Sepsis	79.5	17.8	45.6	50
Shock	61.5	37.8	46.2	53.1
Mechanical ventilation	87.2	4.4	44.2	28.6
Cirrhosis	10.3	86.7	40.0	52.7
History of ulcer	7.7	93.3	50.0	53.8
Acute anemia	61.5	42.2	48.0	55.9
Hyperuremia	5.1	97.8	66.7	54.3
Hemodynamic instability	7.7	100	100	55.6
Digestive bleeding	25.6	64.4	38.5	50.0
Vomiting	15.4	82.2	42.9	52.9

Data are presented as %

*PPV* positive predictive value, *NPV* negative predictive value

## Discussion

In our 6-year retrospective review of all UGE performed in a single medico-surgical ICU, we showed that although regularly performed, UGE for critically ill patients initially hospitalized for another reason than upper digestive bleeding but for whom the question of upper digestive bleeding is raised during their ICU stay has limited diagnostic and therapeutic interest during ICU stay.

This is, to our knowledge, the largest study assessing the profitability of this procedure in this specific patient population.

In this population of non-selected ICU patients, with the suspicion of ICU-acquired upper digestive bleeding, we found that UGE was strictly normal in one third of procedures. When performed in patients in whom gastrointestinal bleeding was not suspected, Ovenden et al. reported that UGE was normal in two-thirds of procedures [18]. The vast majority of abnormal findings we observed (*n* = 45; 76%) were either peptic lesions (*i.e.,* ulcer or esophagitis/gastritis) or nasogastric tube-associated erosions. Similar lesions were reported by Ovenden et al. In this series of 74 patients who underwent an UGE, a pathological finding was found in 34% of the subjects, either gastritis/erosions in 14%, nasogastric tube trauma in 8 (11%), esophagitis in 4 (5%) and non-bleeding duodenal ulceration in 3 (4%) [18].

In our series, an active bleeding was only retrieved in three of the 13 peptic ulcers at the time of the UGE, therefore requiring an instrumental hemostasis procedure (submucosal adrenaline injection and clip application). Nasogastric tube erosions were always considered incidental findings that could not be held responsible for the symptoms and that did not change the patients’ management. The other lesions found were mostly incidental that did not account for any significant digestive bleeding. We can infer that not only the suspicion of active bleeding is very infrequent, but also, when suspected its occurrence, is very rare.

These results are of importance since UGE is a costly and time-consuming procedure, for both ICU and endoscopy teams, that can cause significant morbidity if performed unduly [21]. Its poor performance can probably be explained by the large prescription of prophylactic proton pump inhibitors in our unit for the patients that present several risk factors for the so-called stress related mucosal disease. Although prevention with prophylactic proton pump inhibitor has been showed to be safe [4, 5, 22], its use is still being challenged [23–25]. Our study population was composed of patients presenting multiple organ failure so the results cannot be explained by a lack of severity of the patients, as attested by the high SOFA score.

Whereas previous reports focused on UGE performed in the ICU for overt gastrointestinal bleeding [7, 16], Plaisier et al. [15] described the indications and results of 411 gastroscopies performed in 4 ICUs of a single Dutch hospital. Unlike our series, most patients were admitted for a gastrointestinal hemorrhage. Nevertheless, in patients undergoing the UGE for another reason, esophagitis, gastritis and gastric ulcer were the most frequent coincidental findings, as in our series. Of interest, in this setting, UGE was also widely used (in 35% of cases) for the placement of feeding tubes, which was a very uncommon indication in our series.

The operative values of the symptoms and conditions that justified performing the UGE are disappointingly poor. None of them was found to be discriminating enough to be useful, alone, in clinical practice.

It is interesting to note that we observed an unexpectedly high number of esophageal candidiasis. None of these patients had an HIV infection or hematologic malignancy, and this diagnosis was never evoked before the UGE was performed. Although this was considered an incidental finding and an unlikely cause of digestive bleeding, all of them were treated by fluconazole [26, 27]. Risk factors and incidence of esophageal candidiasis in ICU patients are poorly studied [27], and our work raises the concern that this condition might be underdiagnosed and undertreated.

Our study holds several limitations. First, the retrospective design does not allow to drawing any definitive conclusion concerning the efficacy of upper digestive endoscopy in ICU patients suspected of ICU-acquired upper digestive bleeding. Nevertheless, the review of the whole charts and the gastroscopy report of the patients included enables to retrace the exact motives of the endoscopy.

Second, although we do acknowledge the number of patients is small (*n* = 84) with regard to the number of subjects admitted in our ICU during the study period (*n* = 3352), we can explain it with our policy of proton pump inhibitor prescription in subjects with a risk factor for digestive bleeding, and of active enteral nutrition of all patients for whom the digestive tract can be used. Third, our data reflect the experience of a single center, and the decision to perform the endoscopies or the hemostatic procedures were left to the attending intensivist and endoscopist and we cannot rule out that the results might have been different in another patient population treated by another medical team and our findings may not be generalized. Nevertheless, the decision to perform a gastroscopy is generally taken within the whole ICU medical team, with habits that did not change during the study period, and hemostatic procedures were performed according to the standard guidelines.

These limitations taken into account, we propose that UGE is of very limited use in ICU patients suspected of ICU-acquired upper digestive bleeding. The low yield of UGE in our center suggests that these patients can be managed with a watchful waiting when hemodynamically stable.

## Conclusions

Bedside UGE has very poor diagnostic and therapeutic performances when performed in a population of intensive care patient suspected of ICU-acquired upper digestive bleeding. These results should be confirmed by a prospective multicenter observational cohort.

## Abbreviations

ICU: Intensive care unit
SOFA: Sequential Organ Failure Assessment
UGE: upper gastrointestinal endoscopy
